# Induction of JNK and c-Abl signalling by cisplatin and oxaliplatin in mismatch repair-proficient and -deficient cells

**DOI:** 10.1038/sj.bjc.6690176

**Published:** 1999-03

**Authors:** A Nehmé, R Baskaran, S Nebel, D Fink, S B Howell, J Y J Wang, R D Christen

**Affiliations:** Department of Medicine and the Cancer Center, University of California, 9500 Gilman Drive, La Jolla, CA 92093-0058, USA; Department of Biology and Center for Molecular Genetics, University of California, La Jolla, San Diego, CA 92093-0322, USA

**Keywords:** cisplatin, oxaliplatin, JNK/SAPK, c-Abl, DNA mismatch repair

## Abstract

Loss of DNA mismatch repair has been observed in a variety of human cancers. Recent studies have shown that loss of DNA mismatch repair results in resistance to cisplatin but not oxaliplatin, suggesting that the mismatch repair proteins serve as a detector for cisplatin but not oxaliplatin adducts. To identify the signal transduction pathways with which the detector communicates, we investigated the effect of loss of DNA mismatch repair on activation of known damage-responsive pathways, and recently reported that cisplatin differentially activates c-Jun NH2-terminal kinase (JNK) and c-Abl in repair-proficient vs.-deficient cells. In the current study, we directly compared differential activation of these pathways by cisplatin vs. oxaliplatin. The results confirm that cisplatin activates JNK kinase 5.7 ± 1.5 (s.d.)-fold more efficiently in DNA mismatch repair-proficient than repair-deficient cells, and that the c-Abl response to cisplatin is completely absent in DNA mismatch repair-deficient cells. In contrast, there was no detectable activation of the JNK or c-Abl kinases in DNA mismatch repair-proficient or -deficient cells exposed to oxaliplatin. The present study demonstrates that, despite the similarity of the adducts produced by cisplatin and oxaliplatin, they appear to be recognized by different detectors. The DNA mismatch repair system plays an important part in the recognition of cisplatin adducts, and activation of both the JNK and c-Abl kinases in response to cisplatin damage is dependent on the detector function of the DNA mismatch repair proteins. In contrast, this detector does not respond to oxaliplatin adducts. © 1999 Cancer Research Campaign


*British Journal of Cancer* (1999) **79**(7/8), 1104–1110
© 1999 Cancer Research Campaign
Article no. bjoc.1998.0176

### Induction of JNK and c-Abl signalling by cisplatin and oxaliplatin in mismatch repair-proficient and -deficient cells

**A Nehmé[1], R Baskaran[2], S Nebel[1], D Fink[1], SB Howell[1], JYJ Wang[2] and RD Christen[1]**

[1]Department of Medicine and the Cancer Center, University of California, San Diego, 9500 Gilman Drive, La Jolla, CA 92093-0058; [2]Department of Biology and Center for Molecular Genetics, University of California, San Diego, La Jolla, CA 92093-0322 USA

**Summary** Loss of DNA mismatch repair has been observed in a variety of human cancers. Recent studies have shown that loss of DNA mismatch repair results in resistance to cisplatin but not oxaliplatin, suggesting that the mismatch repair proteins serve as a detector for cisplatin but not oxaliplatin adducts. To identify the signal transduction pathways with which the detector communicates, we investigated the effect of loss of DNA mismatch repair on activation of known damage-responsive pathways, and recently reported that cisplatin differentially activates c-Jun NH2-terminal kinase (JNK) and c-Abl in repair-proficient vs.-deficient cells. In the current study, we directly compared differential activation of these pathways by cisplatin vs. oxaliplatin. The results confirm that cisplatin activates JNK kinase 5.7 $\pm$ 1.5 (s.d.)-fold more efficiently in DNA mismatch repair-proficient than repair-deficient cells, and that the c-Abl response to cisplatin is completely absent in DNA mismatch repair-deficient cells. In contrast, there was no detectable activation of the JNK or c-Abl kinases in DNA mismatch repair-proficient or -deficient cells exposed to oxaliplatin. The present study demonstrates that, despite the similarity of the adducts produced by cisplatin and oxaliplatin, they appear to be recognized by different detectors. The DNA mismatch repair system plays an important part in the recognition of cisplatin adducts, and activation of both the JNK and c-Abl kinases in response to cisplatin damage is dependent on the detector function of the DNA mismatch repair proteins. In contrast, this detector does not respond to oxaliplatin adducts.

**Keywords:** cisplatin; oxaliplatin; JNK/SAPK; c-Abl; DNA mismatch repair

The platinum-based compound cisplatin is among the most widely used and effective anti-cancer drugs currently available (Kelland, 1993). However, its activity is limited by the fact that many tumours are intrinsically resistant or develop resistance during therapy, and by its neuro- and nephrotoxicity. Several platinum analogues that are able to circumvent cisplatin resistance, and/or the severe side-effects of cisplatin, are currently under clinical evaluation (Christian, 1992). Oxaliplatin is one of the 1,2-diaminocyclohexane (DACH) carrier ligand-based groups of platinum drugs (Chaney, 1995). Like other DACH-based platinum compounds, oxaliplatin has shown lack of cross-resistance in several cisplatin-resistant tumour cell lines (Los et al, 1990; Schmidt and Chaney, 1993). Cytotoxicity of platinum drugs is believed to result from the formation of platinum–DNA adducts. Eukaryotic cells respond to the presence of cisplatin and oxaliplatin adducts in DNA by activating signal transduction pathways that result in cell cycle arrest, an increase in DNA repair activity and apoptosis (Eastman, 1990; Woynarowski et al, 1997). There are no data available on what signalling pathways are activated by oxaliplatin, and only few data with respect to cisplatin. Recent studies have shown that the cisplatin-induced injury response involves activation of the stress-activated protein kinase (SAPK, also known as c-Jun NH2-terminal kinase or JNK) and the nuclear c-Abl protein tyrosine (Liu et al, 1996*a*; Kharbanda et al, 1997; Nehme et al, 1997). The JNK kinases are a family of p54/p46 serine threonine kinases that are related to the MAP kinase family (Derijard et al, 1995). JNK kinases phosphorylate the three nuclear transcription factors, including c-Jun, ATF2 and Elk-1, and stimulate their transcriptional activities (Hibi et al, 1993; Cavigelli et al, 1995; Gupta et al, 1995). The product of the c-*abl* gene is a non-receptor tyrosine kinase that shares structural features with the Src family of tyrosine kinases (Wang, 1993). The SH3 domain of c-Abl binds to DNA-dependent protein kinase (Kharbanda et al, 1997) and RNA polymerase II (Baskaran et al, 1996), which have been identified as c-Abl substrates. Activation of the JNK and c-Abl kinases by DNA damage may constitute an initial step in the generation of signals that coordinate the full injury response.

Loss of DNA mismatch repair has been observed in a wide variety of tumours (Orth et al, 1994). These cancers include most tumours associated with hereditary non-polyposis colorectal cancer (HNPCC), as well as a significant fraction of sporadic colon, gastric, pancreatic, endometrial, ovarian and small-cell lung carcinomas. The genes that encode proteins with roles in DNA mismatch repair in humans include the MutS homologues hMSH2, hMSH3, hMSH6 (GTBP/p160) and the MutL homologues hMLH1, hPMS1 and hPMS2 (Kolodner, 1996; Fishel and Wilson, 1997).

We have previously reported on the drug sensitivities of several pairs of cell lines molecularly engineered to differ with respect to DNA mismatch repair capacity. The hMLH1-deficient HCT116 and HCT116+ch2 cells were twofold resistant to cisplatin compared with the repair-proficient subline HCT116+ch3 that expresses a wild-type copy of *hMLH1* (Aebi et al, 1996; Fink et al,

*Received 28 April 1998*
*Revised 23 June 1998*
*Accepted 25 June 1998*

*Correspondence to:* RD Christen, Department of Medicine 0058, University of California San Diego, 9500 Gilman Drive, La Jolla, CA 92093-0058 USA

1996). A difference of the same magnitude was observed between the HEC59 DNA mismatch repair-deficient cells and the proficient HEC59+ch2 subline that expresses a wild-type copy of *hMSH2*. However, for neither cell pair was there a difference in sensitivity to oxaliplatin (Fink et al, 1996). The difference in cisplatin sensitivity was not due to a difference in the amount of drug taken up into the cells or the extent of DNA platination after a 1-h exposure to 100 μM cisplatin (Aebi et al, 1997). It was also not due to a difference in the ability of cisplatin to induce expression of p53 (Nehme et al, 1997). However, cisplatin activated JNK kinase more efficiently in DNA mismatch repair-proficient than in repair-deficient cells, and activation of c-Abl was completely absent from the DNA mismatch repair-deficient cells (Nehme et al, 1997). In contrast with the situation for cisplatin, loss of DNA mismatch repair due to deficiency of either hMLH1 or hMSH2, was found not to produce resistance to oxaliplatin (Fink et al, 1996; Fink et al, 1997). Failure of loss of DNA mismatch repair to cause resistance to oxaliplatin appeared to be linked to failure of the DNA mismatch repair proteins to recognize this type of adduct and form complexes on platinated DNA in gel shift assays (Fink et al, 1996).

It is our hypothesis that the DNA mismatch repair proteins serve as a detector for cisplatin but not oxaliplatin adducts, because loss of mismatch repair function results in resistance to the former but not the latter. In order to serve as a detector, the mismatch repair system must be able to initiate activation of proapoptotic signalling pathways. Indeed, hMSH2, either by itself (Mello et al, 1996) or in conjunction with hMSH6 (Duckett et al, 1996), has been shown to bind to DNA platinated by cisplatin. If this hypothesis is correct, it should be possible to identify the signal transduction pathways with which the detector communicates by comparing pathways differentially activated during the cisplatin- but not oxaliplatin-induced cellular injury response in mismatch repair-proficient and -deficient cells. Therefore, we investigated the effect of loss of DNA mismatch repair on the activation of the JNK and c-Abl kinases in response to cisplatin- and oxaliplatin-induced cellular injury. We report here that, unlike cisplatin, oxaliplatin does not produce differential activation of JNK and c-Abl kinases in DNA mismatch repair-proficient and -deficient cells.

### METHODS

### Cell lines and culture

The *hMLH1*-deficient human colorectal adenocarcinoma cell line HCT116 was obtained from the American Type Culture Collection (ATCC CCL 247, Rockville, MD, USA), a subline into which a wild-type copy of *hMLH1* on chromosome 3 had been introduced by microcell fusion (HCT116+ch3) and a subline that received chromosome 2 as a negative control (HCT116+ch2) were obtained from Drs C.R. Boland and M. Koi (Koi et al, 1994). The *hMSH2*-deficient human endometrial adenocarcinoma cell line HEC59 (Umar et al, 1994) and a subline complemented with chromosome 2 containing a wild-type copy of *hMSH2* (HEC59 + ch2) were also obtained from Dr C.R. Boland and M. Koi (Umar et al, 1997). All cell lines were maintained in Iscove's modified Dulbecco medium (Irvine Scientific, Irvine, CA, USA) supplemented with 100 mM L-glutamine and 10% heat-inactivated fetal bovine serum. The chromosome-complemented lines were grown in medium supplemented with geneticin (400 μg $ml^{-1}$ for HCT116+ch2 and HCT116+ch3, and 600 μg $ml^{-1}$ for HEC59 + ch2) (Gibco BRL, Gaithersburg, MD, USA). Western blot was used to document expression of hMLH1 and hMSH2 proteins in each of the cell lines (data not shown). The *c-abl*-null mouse fibroblasts (3T3-$Abl^{-/-}$), the polyclonal populations of reconstituted 3T3-$Abl^{+}$ cells and the 3T3 mock infected cells (3T3-mock) were obtained from Dr JYJ Wang (Liu et al, 1996*a*). The 3T3-$Abl^{+}$ and 3T3-mock cells were obtained, respectively, by infection and selection of 3T3-$Abl^{-/-}$ cells with *c-abl* or empty recombinant retroviruses containing the hygromycin resistance gene. The 3T3-mock cells were used as a negative control in all the experiments. All cell lines were maintained in Dulbecco's modified Eagle medium (Irvine Scientific) supplemented with 100 mM L-glutamine and 10% heat-inactivated fetal bovine serum. The absence or presence of expression of c-Abl in 3T3-$Abl^{-/-}$, 3T3-mock, and 3T3-$Abl^{+}$ fibroblasts was verified by immunoblot analysis (data not shown).

### Reagents

Oxaliplatin was a gift from Debiopharm (Lausanne, Switzerland); a stock solution of 1 mM was prepared in water and kept at –20°C. Cisplatin was obtained from Bristol–Myers Squibb Co., Princeton, NJ, USA; a stock solution of 1 mg $ml^{-1}$ was prepared in 0.9% sodium chloride.

### Immunoprecipitations

Cells growing in log phase were treated with cisplatin for 1 h at their respective $IC_{90}$ concentrations (25 μM for the HCT116 cells and 14 μM for the HEC59 cells), or with oxaliplatin for 1 h at their $IC_{90}$ concentrations (30 μM for the HCT116 and HEC59 cells). At 2 h after the beginning of the drug exposure, cells were lysed in 1 ml of lysis buffer (10 mM Tris-HCl, pH 7.4, 150 mM sodium chloride, 5 mM EDTA, 1% Triton X-100, 5 mM dithiothreitol, 1 mM sodium vanadate, 0.1 mM phenylmethylsulphonyl fluoride and 5 mM aminocaproic acid) for 30 min on ice. The insoluble material was removed by centrifugation at 30 000 ***g*** for 20 min at 4°C. Equal amounts of protein were incubated with protein A-Sepharose and anti-JNK1 (2.5 μg) (C-17, Santa Cruz Biotechnology, Santa Cruz, CA, USA), or anti-Abl (2.5 μg) (K-12, Santa Cruz Biotechnology).

### JNK1 kinase assay

The method described by Derijard et al (1994) was used to determine JNK1 kinase activity. Anti-JNK1 immune complexes were washed twice with lysis buffer and then once with kinase buffer (20 mM Hepes, pH 7.0, 10 mM magnesium chloride, 10 mM manganese chloride, 2 mM DTT and 0.1 mM sodium vanadate). Immune complexes were then resuspended in 30 μl of kinase buffer containing 5 μCi [$\gamma$-$^{32}$P]ATP (3000 Ci $mmol^{-1}$; NEN, Boston, MA, USA), 20 μM cold ATP, and 2.5 μg of glutathione S-transferase (GST)-c-Jun (1–79) as substrate (Santa Cruz Biotechnology) and incubated for 20 min at 30°C. To terminate reaction, sodium dodecyl sulphate (SDS) sample butter was added and samples were boiled for 5 min. The phosphorylated proteins were resolved by 10% SDS-PAGE and visualized by autoradiography. Anti-JNK1 immunoprecipitates were examined for their JNK1 content by immunoblotting with JNK1 antibody (C-17, Santa Cruz Biotechnology). The antigen–antibody complexes were visualized by enhanced chemiluminescence (Amersham, Arlington Heights, IL, USA).

© Cancer Research Campaign 1999

### c-Abl kinase assay

The method described by Baskaran et al (1996) was used to determine c-Abl kinase activity. In brief, anti-Abl immune complexes were washed with lysis buffer/500 mM sodium chloride, lysis buffer/100 mM sodium chloride and lysis buffer alone and then washed twice with kinase buffer containing (25 mM Tris-HCl, pH 7.4, 10 mM magnesium chloride, and 1 mM DTT). The washed pellet was resuspended in 20 µl of kinase buffer with 50 µCi of [$\gamma$-$^{32}$P]ATP (3000 Ci mmol$^{-1}$; NEN), 10 µM cold ATP, and 1 µg of GST-CTD as substrate and incubated for 30 min at room temperature. Reactions were terminated by the addition of the SDS sample buffer and boiling for 5 min. Phosphoproteins were fractionated by 5–15% SDS-PAGE and visualized by autoradiography. The amounts of c-Abl protein in the anti-Abl immunoprecipitates were determined by immunoblotting with anti-Abl (8E9, Pharmingen, San Diego, CA, USA). The antigen–antibody complexes were visualized by enhanced chemiluminescence.

### Cytotoxicity assays

3T3-Abl$^{-/-}$, 3T3-mock, and 3T3-Abl$^{+}$ mouse fibroblasts do not form discrete colonies. Therefore, sulphorhodamine B growth rate assays were performed by seeding fibroblasts into 96-well plates at a density of 1500 cells per well in 100 µl of medium. After 24 h, appropriate concentrations of cisplatin or oxaliplatin were added in a final volume of 100 µl of medium, and the cells were exposed for 1 h. Thereafter, the drug-containing medium was aspirated and new drug free medium was added. After 72 h, growth was stopped by adding 50 µl of 50% (w/v) trichloroacetic acid, and cellular protein was stained with sulphorhodamine B and was measured by spectrophotometry (Skehan et al, 1990). Control plates were fixed to estimate the cellular protein at time 0 ($T_0$). The relative growth rate $R$ was calculated as previously reported (Monks et al, 1991). If $T \geq T_0$, $R = (T - T_0)/(C - T_0)$; if $T < T_0$, $R = (T - T_0/T_0$; with $T$ being the absorbance at 72 h in control untreated wells, and $T_0$ being the absorbance in control wells measured immediately before drug treatment. Each experiment was performed in triplicate, and $IC_{50}$ values were estimated by linear interpolation at $r = 0.5$. We have shown that the optimal seeding density was 1500 cells per well, which resulted in an exponentially growing, subconfluent cell layer at 72 h after seeding. The fibroblasts used in these studies have a doubling time of approximately 24 h. Preliminary experiments indicated that an incubation time of 3 days, corresponding approximately to three cell doublings, resulted in reproducible treatment-induced changes in cell growth.

### RESULTS

### Effect of cisplatin and oxaliplatin on JNK1 activity in HCT116 cells

Figure 1 shows the structures of cisplatin and oxaliplatin. During intracellular aquation, the chloride groups are released from cisplatin and the oxalate group from oxaliplatin. Following reaction with DNA, the adducts differ by virtue of the fact that the cisplatin adduct has two amino groups projecting into the major groove whereas the oxaliplatin adduct retains the bulkier DACH ring. The major adducts formed by oxaliplatin are the Pt-GG and Pt-AG intrastrand cross-links (Saris et al, 1996). Sedimentation analysis showed that oxaliplatin does not induce detectable interstand cross-links but forms DNA strand breaks (Woynarowski et al, 1997). In contrast, cisplatin produces interstand crosslinks but no breaks. Oxaliplatin appears to be less reactive with cellular DNA than cisplatin, but is a comparably potent inhibitor of DNA and protein synthesis (Saris et al, 1996; Woynarowski et al, 1997).

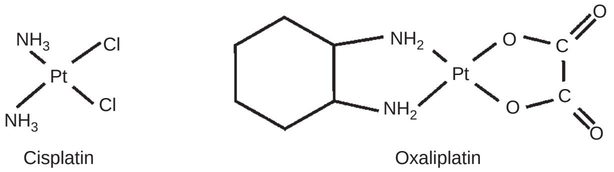

**Figure 1** Structures of cisplatin and oxaliplatin

We have previously shown that cisplatin activates JNK kinase more efficiently in DNA mismatch repair-proficient than repair-deficient cells (Nehme et al, 1997). We have now conducted a new set of experiments to directly compare the effects of cisplatin and oxaliplatin on JNK1 activity. HCT116+ch2 and HCT116+ch3 cells were exposed to 25 µM cisplatin or 30 µM oxaliplatin for 1 h, and lysates prepared at 2 h after the beginning of drug exposure were precipitated with anti-JNK1 antibody. GST-c-Jun (1–79) was used as a substrate to determine the kinase activity of immune complexes (Derijard et al, 1994). A low level of GST-Jun phosphorylation was detectable in the anti-JNK1 immunoprecipitates obtained from untreated cells (Figure 2, lanes 1 and 4). In accordance with our previous finding (Nehme et al, 1997), cisplatin activated JNK1 kinase more efficiently in the repair-proficient subline HCT116+ch3 [8.6 ± 2.5 (s.d.)-fold activation] than in the repair-deficient HCT116+ch2 subline [1.5 ± 1.1 (s.d.)-fold activation] (Figure 2, lanes 2 and 5). However, oxaliplatin failed to activate JNK1 in either DNA mismatch repair-proficient or -deficient HCT116 cells (Figure 2, lanes 3 and 6). In three separate experiments, neither cisplatin nor oxaliplatin induced significant changes in JNK1 protein levels. Therefore, the increase in activity induced by cisplatin was due to activation of the enzyme because there was no significant effect of cisplatin on JNK1 protein level.

### Effect of cisplatin and oxaliplatin on JNK1 activity in HEC59 cells

A second pair of mismatch repair-deficient (HEC59) and -proficient (HEC59+ch2) cell lines was used to further compare the activation of JNK1 by cisplatin and oxaliplatin. We have previously shown that cisplatin failed to activated JNK1 activity in mismatch repair-proficient and -deficient HEC59 cells (Nehme et al, 1997). We directly compared the effects of cisplatin and oxaliplatin on JNK1 activity in HEC59 cells. In this cell system, there was no detectable activation of JNK1 after cisplatin or oxaliplatin treatment (Figure 2, lanes 7–12). Moreover, cisplatin and oxaliplatin had no detectable effect on JNK1 levels (Figure 2). These results suggest that the response to these two drugs is defective in both deficient and proficient HEC59 cells. UV-C light, a potent inducer of JNK1 (Derijard et al, 1994), did produce activation of JNK1 kinase in both types of HEC59 cells (data not shown), indicating that JNK1 is functionally expressed but not activated by cisplatin or oxaliplatin in the HEC59 cells.

### Effect of cisplatin and oxaliplatin on c-Abl activity in HCT116 cells

The observation that cisplatin activates the nuclear c-Abl tyrosine kinase has been documented in several recent reports (Kharbanda et

 © Cancer Research Campaign 1999

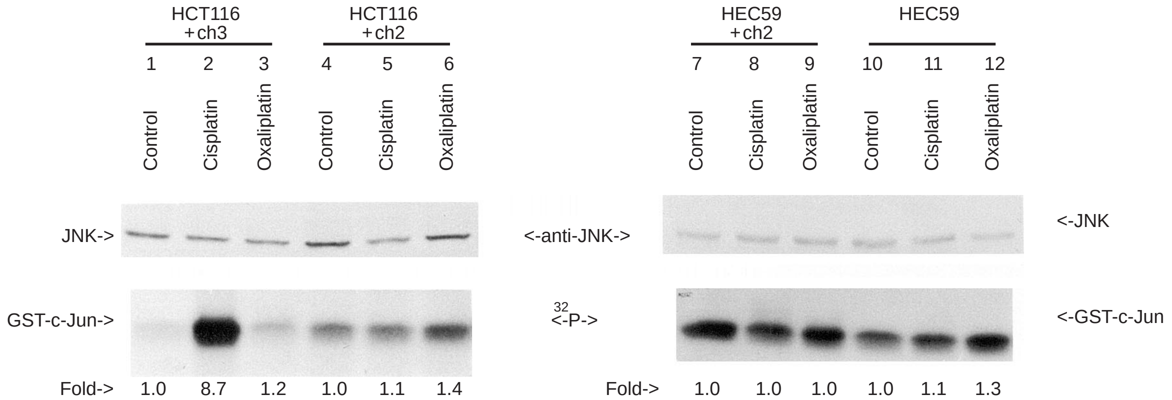

**Figure 2** Effect of cisplatin and oxaliplatin on JNK1 activity. Cells were exposed to cisplatin (25 μM for the HCT116+ch2 and HCT116+ch3 cells, and 14 μM for the HEC59 and HEC59+ch2 cells), or to oxaliplatin (30 μM for the HCT116 and HEC59 cells) for 1 h. At 2 h after the beginning of drug exposure, JNK1 kinase was assayed by immune complex kinase assays with GST-c-Jun (1–79) as a substrate. Immune complexes were examined for their JNK content by immunoblotting with JNK antibody (top panel) or used for GST-c-Jun phosphorylation (bottom panel). The experiment was repeated three times and similar results were obtained

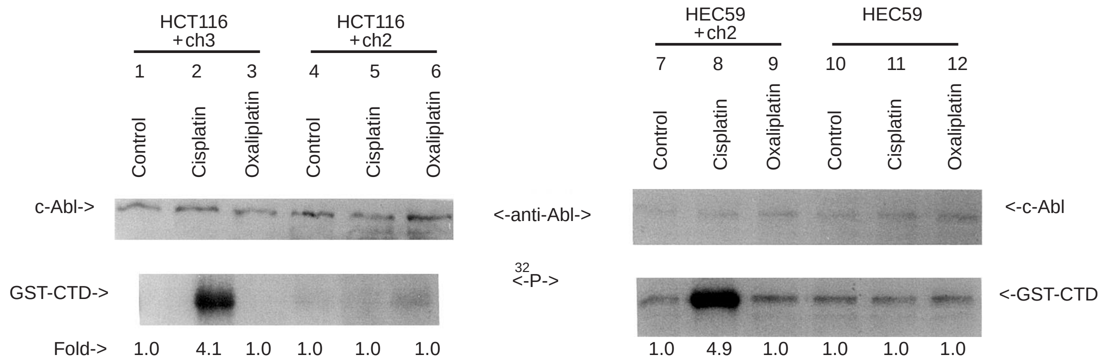

**Figure 3** Effect of cisplatin and oxaliplatin on c-Abl activity. Cells were exposed to cisplatin (25 μM for the HCT116+ch2 and HCT116+ch3 cells, and 14 μM for the HEC59 and HEC59+ch2 cells), or to oxaliplatin (30 μM for the HCT116 and HEC59 cells) for 1 h. At 2 h after the beginning of drug exposure, c-Abl kinase was assayed by immune complex kinase assays with GST-CTD as a substrate. Immune complexes were examined for their c-Abl content by immunoblotting with c-Abl antibody (top panel) or used for GST-CTD phosphorylation (bottom panel). The experiment was repeated three times and similar results were obtained al, 1995; Liu et al, 1996*a*), and, as cisplatin fails to activate JNK in c-Abl-deficient cells, the latter would appear to be located upstream of JNK in this injury response pathway (Kharbanda et al, 1995). We have previously shown that cisplatin activates c-Abl in mismatch repair-proficient but not in mismatch repair-deficient cells (Nehme et al, 1997). We compared the effect of cisplatin and oxaliplatin directly on c-Abl kinase activity. HCT116+ch2 and HCT116+ch3 cells were treated with 25 μM cisplatin or 30 μM oxaliplatin for 1 h, and anti-Abl immunoprecipitates were prepared from cell lysates at 2 h. In vitro kinase assays were performed with the GST-CTD protein as substrate (Baskaran et al, 1996). A low level of GST-CTD phosphorylation was detectable in the untreated cells (Figure 3, lanes 1 and 4), and the extent of GST-CTD phosphorylation increased by a factor of 4.1 ± 0.3 (s.d.)-fold at 2 h after the beginning of cisplatin exposure in the HCT116+ch3 proficient cells (Figure 3, lane 2). In contrast, there was no induction of c-Abl kinase activity in the mismatch repair-deficient HCT116+ch2 cells exposed to cisplatin (Figure 3, lane 5). More importantly, there was no detectable induction of GST-CTD phosphorylation when the HCT116 DNA mismatch repair-proficient and -deficient cells were exposed to oxaliplatin (Figure 3, lanes 3 and 6). The finding that cisplatin had no detectable effect on c-Abl protein level indicated an increase in c-Abl activity (Figure 3, lane 2).

### Effect of cisplatin and oxaliplatin on c-Abl activity in HEC59 cells

The cisplatin- and oxaliplatin-induced c-Abl tyrosine kinase activity was also studied in the HEC59 and HEC59+ch2 cells. Exposure of the mismatch repair-proficient HEC59+ch2 cells to cisplatin produced a 4.5 ± 0.4 (s.d.)-fold stimulation of c-Abl kinase activity at 2 h after the beginning of drug exposure (Figure 3, lane 8). However, there was no detectable induction of GST-CTD phosphorylation when the HEC59 DNA mismatch repair-deficient cells were exposed to cisplatin (Figure 3, lane 11). Oxaliplatin failed to induce GST-CTD phosphorylation in either the DNA mismatch repair-proficient or -deficient HEC59 cells (Figure 3, lanes 9 and 12). Immunoblot analysis with anti-Abl antibody showed no detectable effect of cisplatin or oxaliplatin on c-Abl levels in either the HEC59 or HEC59+ch2 cell lines (Figure 3).

© Cancer Research Campaign 1999

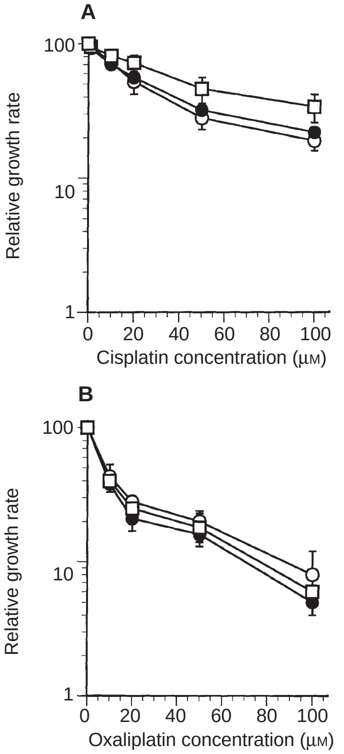

**Figure 4** Effect of the loss of c-Abl kinase on sensitivity to cisplatin (**A**) and oxaliplatin (**B**). 3T3-Abl$^{-/-}$(□), 3T3-mock (○), and 3T3-Abl$^{+/+}$ (●) cells were treated with appropriate concentrations of cisplatin or oxaliplatin for 1 h. The cisplatin- and oxaliplatin-induced growth inhibition was determined by using the sulphorhodamine B growth rate assay. Each data point represents the mean of five independent experiments performed with triplicate culture, bars represent standard deviations

### Effect of c-Abl expression on sensitivity to cisplatin and oxaliplatin

We investigated the effect of loss of c-Abl kinase expression on cisplatin and oxaliplatin-induced growth inhibition using 3T3 cells in which both c-*abl* alleles had been knocked out (3T3-Abl$^{-/-}$) and sublines molecularly engineered to express a wild-type copy of c-Abl (3T3-Abl$^{+}$) or mock infected as a control (3T3-mock). Figure 4 shows the relative growth rate as a function of drug concentration for cisplatin and oxaliplatin for the three mouse fibroblast cell lines. The results demonstrate that there was no difference in sensitivity between the *c-abl*-null 3T3-mock cells and the reconstituted 3T3-Abl$^{+}$ cells for cisplatin ($IC_{50}$ 21.4 ± 0.8 for 3T3-mock cells and 22.7 ± 1.5 for 3T3-Abl$^{+}$ cells). Similar results were obtained with oxaliplatin ($IC_{50}$ 8.5 ± 1.2 for 3T3-mock cells and 8.3 ± 0.5 for 3T3-Abl$^{+}$ cells).

### DISCUSSION

We have previously shown that activation of JNK and c-Abl by cisplatin is dependent on the integrity of DNA mismatch repair function (Nehme et al, 1997). The hypothesis is that DNA mismatch repair proteins serve as a detector for DNA damage due to cisplatin but not oxaliplatin (Hawn et al, 1995; Kat et al, 1995); resistance to cisplatin is linked to the failure of DNA mismatch repair-deficient cells to recognize cisplatin adducts and to activate signalling pathways that eventually trigger apoptosis. If this hypothesis is correct, then the detector function of the mismatch repair proteins must be able to initiate activation of signal transduction pathways in response to cisplatin but not oxaliplatin. The present work identifies JNK1 and c-Abl as members of pathways activated by the detector function of the mismatch repair proteins in response to cisplatin- but not oxaliplatin-induced cellular injury.

In the *hMLH1*-defective HCT116 colon cell model, cisplatin caused a 5.7-fold greater increase in JNK1 activity in the mismatch repair-proficient than in the -deficient cells. In contrast, oxaliplatin did not activate JNK1 kinase in either DNA mismatch repair-proficient or -deficient cells. Cisplatin and oxaliplatin failed to activate JNK1 in either the repair-proficient or -deficient HEC59 endometrial cells, so that a differential effect could not be further documented in a second mismatch repair-proficient and -deficient cell pair. Nevertheless, the data from the HCT116 mismatch repair-proficient and -deficient cells argue that, in cells in which the JNK1 pathway is triggered, mismatch repair activity is required for maximal activation in response to cisplatin- but not oxaliplatin-induced injury. In the case of c-Abl, cisplatin produced a 4.1- and 4.5-fold increase in activity in the proficient cells of both pairs (HCT116+ch3 and HEC59+ch2 cells), that was completely ablated by loss of DNA mismatch repair function in both types of cells (HCT116+ch2 and HEC59). Thus, cisplatin consistently activated c-Abl and this activation was consistently mismatch repair dependent in the two pairs of cell lines examined. In contrast, oxaliplatin failed to activate c-Abl irrespective of the mismatch repair status of the cells. The biochemical mechanisms that link DNA mismatch repair proteins to the activation of the JNK1 and c-Abl pathways have yet to be identified. Both JNK1 and c-Abl have established nuclear functions and places these molecules in a position where they can provide such coupling independent of the activation status of cytoplasmic mitogen activated protein kinases (Kipreos and Wang, 1992; Cavigelli et al, 1995).

The failure of cisplatin to activate JNK at concentrations that produced an increase in c-Abl activity in the HEC59 cell system suggests that these kinases are not part of the same signalling pathway, and that JNK1 activation is not a universal feature of the cisplatin-induced cellular injury response. These conclusions are consistent with recent observations of Liu et al (1996*a*), who showed that activation of c-Abl and JNK represent distinct signalling responses to DNA damage. Failure of JNK1 activation in HEC59 cells may be related to the fact that these cells exhibit microsatellite instability (Umar et al, 1994), and it is possible that there has been mutational inactivation of some proteins upstream of JNK in the signalling response to cisplatin. Cisplatin and oxaliplatin kill cells by activating apoptosis (Chen et al, 1996; Woynarowski et al, 1997); however, the fact that cisplatin and oxaliplatin failed to activate JNK at concentrations that produced apoptosis in either the HEC59 or HEC59+ch2 cells suggests that JNK activation is not essential to successful initiation of apoptosis by these drugs. This is consistent with recent evidence indicating that JNK is not on the critical pathway by which apoptosis is activated in human breast cancer cells exposed to TNF (Liu et al, 1996*b*). No other information is currently available on the role that JNK activation plays in platinum drug-induced apoptosis.

Although the role of the ATM (ataxia telangiectasia mutated) gene product as an upstream activator of c-Abl during the cellular injury response to radiation has recently been elucidated (Baskaran et al, 1997; Shafman et al, 1997), there is limited information about how c-Abl kinase actually triggers apoptosis in response to this type of cellular injury. Recent studies by Yuan et al (1997) have demonstrated that mouse embryonic fibroblasts deficient in

 *© Cancer Research Campaign 1999*

c-Abl kinase are more resistant to the killing effects of ionizing radiation compared with the wild-type cells, although this has not been consistently observed (Liu et al, 1996*a*). The overexpression of c-Abl inhibits growth by causing cell cycle arrest, and this growth-suppressive activity is functionally similar to that of tumour suppressor genes such as p53 and Rb (Sawyers et al, 1994; Mattioni et al, 1995). Despite the fact that cisplatin consistently activates c-Abl, it is not clear that such activation is required for the triggering of apoptosis as the c-abl -null 3T3-mock cells are not significantly resistant to this drug when compared with the reconstituted 3T3-Abl⁺ cells. This is consistent with recent work of Liu et al (1996*a*) who showed that loss of c-Abl does not contribute to increased cisplatin-sensitivity. Thus, it is likely that the mismatch repair system also triggers other pathways more directly coupled to the generation of proapoptotic activity.

### ACKNOWLEDGEMENTS

This work was supported by NIH grant CA43054 (to JYJW), grant 4154 from the Council for Tobacco Research, grants from CaP CURE, the Colleen Gilbert Foundation, and Sanofi Research Inc. This work was conducted in part by the Clayton Foundation for Research – California Division. Drs Howell and Christen are Clayton Foundation Investigators.

### ABBREVIATIONS

CTD, carboxy-terminal repeated domain; DACH, 1,2-diamino-cyclohexane; $IC_{50}$, concentration causing 50% inhibition of cell growth; GST, glutathione S-transferase; JNK, c-Jun $NH_2$-terminal kinase; MAPK, mitogen-activated protein kinase; oxaliplatin, *trans*, 1-1,2-diaminocyclohexane oxalatoplatinum(II); SAPK, stress-activated protein kinase.

### REFERENCES

Aebi S, Kurdi-Haidar B, Gordon R, Cenni B, Zheng H, Fink D, Christen RD, Boland CR, Koi M, Fishel R and Howell SB (1996) Loss of DNA mismatch repair in acquired resistance to cisplatin. *Cancer Res* **56**: 3087–3090

Aebi S, Fink D, Gordon R, Kim HK, Zheng H, Fink JL and Howell SB (1997) Resistance to cytotoxic drugs in DNA mismatch repair-deficient cells. *Clin Cancer Res* **3**: 1763–1767

Baskaran R, Chiang GG and Wang JYJ (1996) Identification of a binding site in c-Abl tyrosine kinase for the C-terminal repeated domain of RNA polymerase II. *Mol Cell Biol* **16**: 3361–3369

Baskaran R, Wood LD, Whitaker LL, Canman CE, Morgan SE, Xu Y, Barlow C, Baltimore D, Wynshaw-Boris A, Kastan MB and Wang JYJ (1997) Ataxia telangiectasia mutant protein activates c-Abl tyrosine kinase in response to ionizing radiation. *Nature* **387**: 516–519

Cavigelli M, Dolfi F, Claret FX and Karin M (1995) Induction of c-fos expression through JNK-mediated TCF/Elk-1 phosphorylation. *EMBO J* **14**: 5957–5964

Chaney SG (1995) The chemistry and biology of platinum complexes with the 1,2-diaminocyclohexane carrier ligand. *Int J Oncol* **6**: 1291–1305

Chen Z, Naito M, Mashima T and Tsuruo T (1996) Activation of actin-cleavable interleukin 1-converting enzyme (ICE) family protease CCP-32 during chemotherapeutic agent-induced apoptosis in ovarian carcinoma cells. *Cancer Res* **56**: 5224–5229

Christian MC (1992) The current status of new platinum analogs. *Semin Oncol* **19**: 720–733

Derijard B, Hibi M, Wu IH, Barrett T, Su B, Deng T, Karin M and Davis RJ (1994) JNK1: a protein kinase stimulated by UV light and Ha-ras that binds and phosphorylates the c-Jun activation domain. *Cell* **76**: 1025–1037

Derijard B, Raingeaud J, Barrett T, Wu IH, Han J, Ulevitch RJ and Davis RJ (1995) Independent human MAP kinase signal transduction pathways defined by MEK and MKK isoforms. *Science* **267**: 682–685

Duckett DR, Drummond JT, Murchie AIH, Readon JT, Sancar A, Lilley DMJ and Modrich P (1996) Human MutSα recognizes damaged DNA base pairs containing $O^6$-methylguanine, $O^4$-methylthymine, or the cisplatin-d(GpG) adduct. *Proc Natl Acad Sci USA* **93**: 6443–6447

Eastman A (1990) Activation of programmed cell death by anticancer agents: cisplatin as a model system. *Cancer Cells* **2**: 275–280

Fink D, Nebel S, Aebi S, Zheng H, Cenni B, Nehmé A, Christen RD and Howell SB (1996) The role of DNA mismatch repair in platinum drug resistance. *Cancer Res* **56**: 4881–4886

Fink D, Zheng H, Nebel S, Norris PS, Aebi S, Lin T-P, Nehmé A, Christen RD, Haas M, Macleod CL and Howell SB (1997) In vitro and In vivo resistance to cisplatin in cells that have lost DNA mismatch repair. *Cancer Res* **53**: 1841–1845

Fishel R and Wilson T (1997) MutS homologs in mammalian cells. *Curr Opin Genet Dev* **7**: 105–113

Gupta S, Campbell D, Derijard B and Davis RJ (1995) Transcription factor ATF2 regulation by the JNK signal transduction pathway. *Science* **267**: 389–393

Hawn MT, Umar A, Carethers JM, Marra G, Kunkel TA, Boland CR and Koi M (1995) Evidence for a connection between the mismatch repair system and the $G_2$ cell cycle checkpoint. *Cancer Res* **55**: 3721–3725

Hibi M, Lin A, Smeal T, Minden A and Karin M (1993) Identification of an oncoprotein-and UV-responsive protein kinase that binds and potentiates the c-Jun activation domain. *Genes Dev* **7**: 2135–2148

Kat A, Thilly WG, Fang W, Longley MJ, Li G and Modrich P (1995) An alkylation–toleration, mutator human cell line is deficient in strand-specific mismatch repair. *Proc Natl Acad Sci USA* **90**: 6424–6428

Kelland LR (1993) New platinum antitumor complexes. *Crit Rev Oncol Hematol* **15**: 191–219

Kharbanda S, Ren R, Pandey P, Shafman TD, Feller SM, Weichselbaum RR and Kufe DW (1995) Activation of the c-Abl tyrosine kinase in the stress response to DNA-damaging agents. *Nature* **376**: 785–788

Kharbanda S, Pandey P, Jin S, Inoue S, Bharti A, Yan Z-M, Weicheselbaum R, Weaver D and Kufe D (1997) Functional interaction between DNA-PK and c-Abl in response to DNA damage. *Nature* **386**: 732–735

Kipreos ET and Wang JYJ (1992) Cell cycle-regulated binding of c-Abl tyrosine kinase to DNA. *Science* **256**: 382–385

Koi M, Umar A, Chauhan DP, Cherian SP, Carethers JM, Kunkel TA and Boland CR (1994) Human chromosome 3 corrects mismatch repair deficiency and microsatellite instability and reduces *N*-methyl-*N′*-nitro-*N*-nitrosoguanidine tolerance in colon tumor cells with homozygous *hMLH1* mutation. *Cancer Res* **54**: 4308–4312

Kolodner R (1996) Biochemistry and genetics of eukaryotic mismatch repair. *Genes Dev* **10**: 1433–1442

Liu Z-G, Baskaran R, Lea-Chou ET, Wood LD, Chen Y, Karin M and Wang JYJ (1996*a*) Three distinct signalling responses by murine fibroblasts to genotoxic stress. *Nature* **384**: 273–276

Liu Z-G, Hsu H, Goeddel DV and Karin M (1996*b*) Dissection of TNF receptor 1 effector functions: JNK activation is not linked to apoptosis while NF-kB activation prevents cell death. *Cell* **87**: 565–576

Los G, Mutsaers PH, Ruevekamp M and McVie JG (1990) The use of oxaliplatin versus cisplatin in intraperitoneal chemotherapy in cancers restricted to the peritoneal cavity in the rat. *Cancer Lett* **51**: 109–117

Mattioni T, Jackson PK, van Huijsduijnen OB-H and Picard D (1995) Cell cycle arrest by tyrosine kinase Abl involves altered early mitogenic response. *Oncogene* **10**: 1325–1333

Mello JA, Acharya S, Fishel R and Essigmann JM (1996) The mismatch-repair protein hMSH2 binds selectivity to DNA adducts of the anticancer drug cisplatin. *Chem Biol* **3**: 579–589

Monks A, Scudiero D, Skehan P, Shoemaker R, Paull K, Vistica D, Hose C, Langley J, Cronise P, Vaigro-Wolff A, Gray-Woodrich M, Campbell H, Mayo J and Boyd M (1991) Feasibility of a high-flux anticancer drug screen using a diverse panel of cultured human tumor cell lines. *J Natl Cancer Inst* **83**: 757–766

Nehme A, Rajasekaran B, Aebi S, Fink D, Nebel S, Cenni B, Wang JYJ, Howell SB and Christen RD (1997) Differential induction of c-Jun NH2-terminal kinase and c-Abl kinase in DNA mismatch repair-proficient and -deficient cells exposed to cisplatin. *Cancer Res* **57**: 3253–3257

Orth K, Hung J, Gazdar A, Bowcock A, Mathis JM and Sambrook J (1994) Genetic instability in human ovarian cancer cell lines. *Proc Natl Acad Sci USA* **91**: 9495–9499

Saris CP, van de Vaart PJM, Rietbroek RC and Blommaert FA (1996) *In vitro* formation of DNA adducts by cisplatin, lobaplatin and oxaliplatin in calf thymus DNA in solution and in cultured human cells. *Carcinogenesis* **17**: 2763–2769

© Cancer Research Campaign 1999 

Sawyers CL, McLaughlin J, Goga A, Havlik M and Witte O (1994) The nuclear tyrosine kinase c-abl negatively regulates cell growth. *Cell* **77**: 121–131

Schmidt W and Chaney SG (1993) Role of carrier ligand in platinum resistance of human carcinoma cell lines. *Cancer Res* **53**: 799–805

Shafman T, Khanna KK, Kedar P, Spring K, Kozlov S, Yen T, Hobson K, Gatei M, Zhang N, Watters D, Egerton M, Shiloh Y, Kharbanda S, Kufe D and Lavin MF (1997) Interaction between ATM protein and c-Abl in response to DNA damage. *Nature* **387**: 520–523

Skehan P, Storeng R, Scudiero D, Monks A, Mcmahon J, Vistica D, Warren JT, Bokesch H, Kenney S and Boyd MR (1990) New colorimetric cytotoxicity assay for anticancer-drug screening. *J Natl Cancer Inst* **82**: 1107–1112

Umar A, Boyer JC, Thomas DC, Nguyen DC, Risinger JI, Boyd J, Ionov Y, Perucho M and Kunkel TA (1994) Defective mismatch repair in extracts of colorectal and endometrial cancer cell lines exhibiting microsatellite instability. *J Biol Chem* **269**: 14367–14370

Umar A, Koi M, Risinger JI, Glaab W, Tindall KR, Kolodner R, Boland CR, Barrett JC and Kunkel TA (1997) Correction of hypermutability, N-methyl-N′-nitro-N-nitrosoguanidine-resistance and defective DNA mismatch repair by introducing chromosome 2 into human tumor cells with mutations in *MSH2* and *MSH6*. *Cancer Res* **57**: 3949–4955

Wang JYJ (1993) Abl tyrosine kinase in signal transduction and cell-cycle regulation. *Curr Opin Genet Dev* **3**: 35–43

Woynarowski JM, Chapman WG, Napier C and Raymond E (1997) Oxaliplatin effects on naked and intracellular DNA. *Proc Am Assoc Cancer Res* **38**: 311

Yuan ZM, Huang Y, Ishiko T, Kharbanda S, Weichselbaum R and Kufe D (1997) Regulation of DNA damage-induced apoptosis by the c-Abl tyrosine kinase. *Proc Natl Acad Sci USA* **94**: 1437–1440

*© Cancer Research Campaign 1999*
